# Methylation Modification in Ornamental Plants: Impact on Floral Aroma and Color

**DOI:** 10.3390/ijms25158267

**Published:** 2024-07-29

**Authors:** Chenchen Xie, Qingyin Tian, Hanruo Qiu, Rui Wang, Lianggui Wang, Yuanzheng Yue, Xiulian Yang

**Affiliations:** 1 Key Laboratory of Landscape Architecture, College of Landscape Architecture, Nanjing Forestry University, Nanjing 210037, China; 13770285628@163.com (C.X.); tqy9818@163.com (Q.T.); qqqhr2001@163.com (H.Q.); wrplant@njfu.edu.cn (R.W.); wlg@njfu.com.cn (L.W.); 2 Co-Innovation Center for Sustainable Forestry in Southern China, Nanjing Forestry University, Nanjing 210037, China

**Keywords:** methylation, floral aroma and color, gene expression, metabolic regulation, ornamental plants

## Abstract

Methylation represents a crucial class of modification that orchestrates a spectrum of regulatory roles in plants, impacting ornamental characteristics, growth, development, and responses to abiotic stress. The establishment and maintenance of methylation involve the coordinated actions of multiple regulatory factors. Methyltransferases play a pivotal role by specifically recognizing and methylating targeted sites, which induces alterations in chromatin structure and gene expression, subsequently influencing the release of volatile aromatic substances and the accumulation of pigments in plant petals. In this paper, we review the regulatory mechanisms of methylation modification reactions and their effects on the changes in aromatic substances and pigments in plant petals. We also explore the potential of methylation modifications to unravel the regulatory mechanisms underlying aroma and color in plant petals. This aims to further elucidate the synthesis, metabolism, and regulatory mechanisms of various methylation modifications related to the aroma and color substances in plant petals, thereby providing a theoretical reference for improving the aroma and color of plant petals.

## 1. Introduction

Methylation modification reactions involve methyl groups (CH_3_-) being transferred from S-adenosylmethionine (SAM) to carbon, nitrogen, oxygen, or sulfur atoms, facilitated by methyltransferases (MTs). This process modifies DNA, RNA, proteins, or small molecules, resulting in methylation product generation along with S-adenosyl-L-homocysteine [1,2]. Simultaneously, it can undergo demethylation through demethylases (DMs), achieving the dynamic reversibility of methylation modifications. Plant methylation modification has a unique and complex system, which is established and maintained by specific MTs. According to the different substrates for methylation, MTs can be divided into DNA MTs, histone MTs, RNA MTs, and *O*-MTs [3]. Methylation modification pervades the entire lifecycle of plants and is closely linked with secondary metabolism and involved in regulating various biological processes within plants, such as the suppression or termination of gene expression, synthesis and repair of proteins, as well as biosynthesis and degradation of metabolites during physiological activities [4].

Petal color and aroma are among the most striking traits of plants. Any alteration to aroma or color can significantly affect pollinator visitation, foraging, and landing behaviors, ultimately indirectly impacting the reproductive capacity of plants [5]. Additionally, the natural constituents responsible for petal color and aroma find broad applications across diverse industries, such as pharmaceuticals, cosmetics, and nutritional supplements [6]. Different methylation modifications, through their unique regulatory networks, affect the formation of plant aroma substances and pigments. For example, DNA methylation regulates the expression levels of pivotal enzyme genes, thus influencing the synthesis and degradation of terpenoids or carotenoids, and it can serve as an epigenetic mark passed on to the next generation, providing a new source of genetic diversity for plants [7].

Given the significant role of methylation in regulating the color and aroma production in plant petals, this review summarizes the regulation of petal color and aroma changes by methylation modifications. It analyzes the function of structural genes in different plant volatile biosynthesis and pigment metabolism pathways regulated by methylation modifications. We hope that this review will help guide further research and unveil the potential application of methylation modifications in the mechanism of the regulation of plant color and aroma, thus providing a reference for improving plant color and aroma characteristics and enhancing plant ornamental value. Moreover, this review proposes a future research direction for the post-modification of both color and aroma by methylation modification.

## 2. Formation Mechanism of Plant Methylation Modification

### 2.1. DNA Methylation

**Figure 1:**
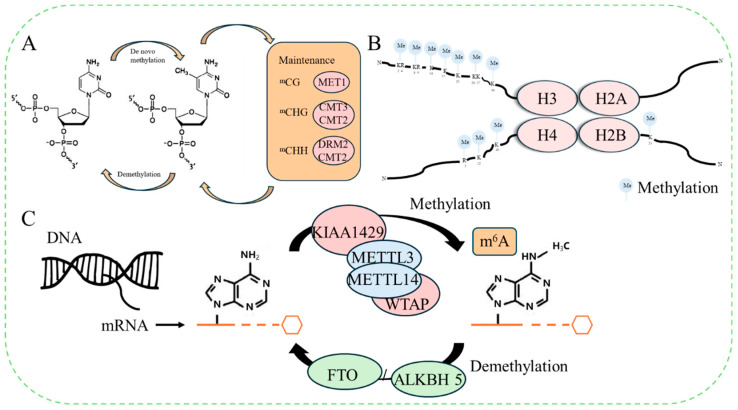
Epigenetic modification of plant methylation. (**A**) DNA methylation; (**B**) histone methylation; (**C**) RNA m^6^A methylation. METTL3/4: methylases; KIAA1429, WTAP, FTO: modified protein; ALKBH5: demethylases.

DNA methylation, a prevalent form of epigenetic alteration, is pivotal in regulating diverse biological functions, including gene transcription, genome integrity maintenance, and transposon suppression. DNA methylation primarily manifests in three distinct sequence contexts in plants: CG, CHG, and CHH (H=T, C, or A). The predominant form of methylation in these contexts is 5-methylcytosine, with minor quantities of N6-methyladenine and 7-methylguanine also existing [8]. Previous studies have shown that gymnosperms contain fewer methylated cytosines in their DNA compared to angiosperms, with approximately 6-30% of cytosines in the genomes of higher plants being methylated [9]. DNA methylation in plants undergoes three dynamic and reversible modification processes, facilitated by DNA MTs (DNMTs): de novo methylation, maintenance methylation, and demethylation [10]. Enzymes responsible for cytosine methylation in plants can be classified into three types: the first includes MTs 1 (MET) responsible for maintaining methylation at CG sites and determining the epigenetic regulation of other related genes [11]; the second comprises plant-specific chromomethylases (CMT), with three CMT genes identified in *Arabidopsis thaliana*, among which *CMT2* and *CMT3* primarily rely on histone H3K9 demethylation to maintain CHG methylation in plants [12]; and the third consists of domain-rearranged MTs (DRM), which catalyze asymmetric cytosine methylation at CHH sites and are primarily responsible for de novo methylation [13] (Figure 1A).

### 2.2. Histone Methylation

Within eukaryotes, a nucleosome is a fundamental repeating unit of chromatin, comprising four core histones (H2A, H2B, H3, and H4) and 147 DNA base pairs [14]. These four core histones all contain two structural domains: the histone fold domain, closely associated with DNA wrapping and histone interactions, and the other located on the periphery of the folded region, where its amino acid residues are catalyzed by various transferases, exhibiting various modification states, such as methylation, acetylation, ubiquitination, and phosphorylation [15]. Histone methylation, a well-studied histone modification, primarily targets specific lysine and arginine residues on histones H3 and H4. It plays diverse roles in regulating gene expression, imprinting, DNA repair, and heterochromatin formation [16]. Depending on the different methylation sites of lysine residues and differences in the number of methyl groups added, chromatin can be endowed with either transcriptional activation or transcriptional repression states. For example, H3K4 and H3K36 are recognized as activated marks, while a series of histone modifications, such as H3K9, H3K27, and H4K20, are deemed to be important inactive marks [17] (Figure 1B).

### 2.3. RNA m^6^A Methylation

RNA m^6^A methylation is a universal modification found in mRNA molecules, mainly affecting biological processes, such as translation, degradation, and the stability of mRNA [18]. The modification is catalyzed by m^6^A MTs, which attach methyl groups to the N6 position of adenine (A) bases within RNA molecules, while demethylation of m^6^A occurs under the catalysis of m^6^A demethylases, erasing the methyl group at this site [19]. In eukaryotic cells, RNA m^6^A is one of the most abundant types of RNA modifications, accounting for 60% of total RNA modifications and participating in multiple stages of RNA metabolism [20]. The m^6^A modification is a dynamic reversible modification formed through the interaction of proteins, such as MTs (“writers”), demethylases (“erasers”), and RNA-binding proteins (“readers”). Genetic maps of m^6^A methylation have been constructed for model plants, like *Oryza sativa* and *Arabidopsis thaliana*, while studies on m^6^A modification epigenomics in other plants are limited [21] (Figure 1C).

### 2.4. O-Methylation

*O*-methylation, catalyzed by *O*-MTs, involve methylation of carboxyl and nitrogen atoms in plant hormones and small molecular compounds, participating in processes, such as chromosome formation and hormone synthesis. In plants, the substrates acted upon by *O*-MTs are diverse, including hormones, chlorophyll, and flavonoids [22,23]. The *O*-MTs can be categorized into three types of small molecule *O*-MTs based on their distinct three-dimensional structures [24]. Type I *O*-MTs facilitate methylation of the oxygen atoms on the hydroxyl groups of phenylpropanoids; for example, isoflavone *O*-MT (IOMT) catalyzes the final step in glycitein biosynthesis by acting on isoflavones in vivo [25]. In *Medicago sativa*, chalcone *O*-MT (ChOMT) is responsible for methylating the 2′-OH of 2′,4,4′-trihydroxychalcone [22]. Type II *O*-MTs use hydroxycinnamic acid coenzyme A esters as catalytic substrates, typically involved in lignin synthesis. For example, caffeoyl coenzyme A 3-*O*-MT (CCoAOMT) exhibits specificity toward coenzyme A-bound phenylpropanoid esters [26]. Type III *O*-MTs mainly catalyze the methylation of small molecule carboxyl and nitrogen atoms in alkaloids, such as theobromine and caffeine. The SABATH family, comprising salicylic acid carboxyl MT (SAMT), benzoic acid carboxyl MT (BAMT), and theobromine synthase (TCS), were among the earliest identified members, and participate in the metabolism of key hormones, like salicylic acid and jasmonic acid (JA) [27].

## 3. Impact of Methylation Modification on the Formation of Floral Aroma in Ornamental Plants

**Figure 2:**
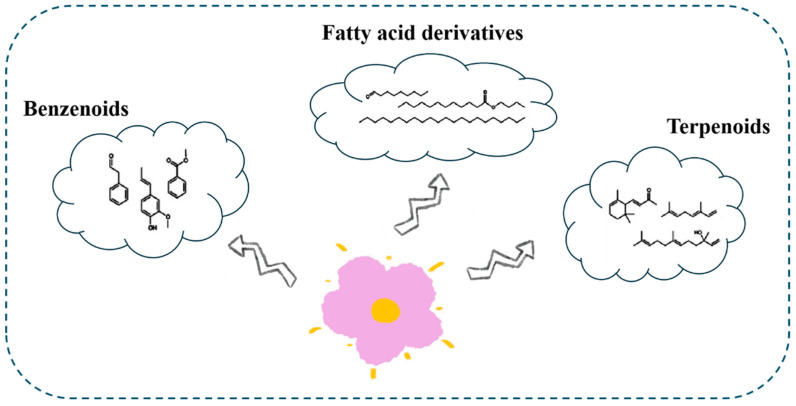
Classification of plant floral substances.

Floral aroma, a key ornamental trait in plants, arises from numerous volatile compounds, and the composition of these compounds varies among plant species and varieties. More than 700 different odor compounds have been identified in plant petals, with the majority being methylated derivatives. This further demonstrates that methylation modifications can influence volatile compounds synthesis [28]. The essence of petal aroma is volatile aromatic substances emitted by plants. The synthesis pathways of plant volatile compounds can be classified into three main categories: terpenoids, phenylpropanoid/benzenoids, and fatty acid derivatives [29] (Figure 2).

### 3.1. Terpenoids

**Figure 3:**
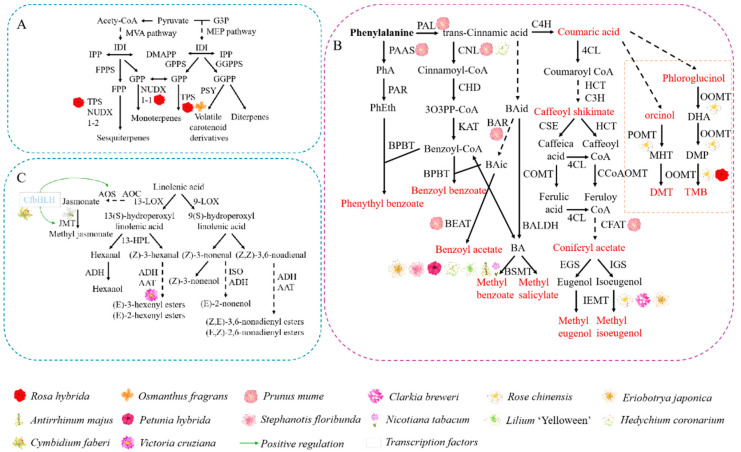
Regulation of aroma synthesis in plant petals. (**A**) Diagram of structural genes involved in the synthesis of terpenoids in flowers; (**B**) diagram of structural genes involved in the synthesis of benzenoids in flowers; (**C**) diagram of structural genes and specific transcription factors involved in the synthesis of fatty acid derivatives in flowers.

Terpenoids, the most abundant class of floral components in plant volatiles, are secondary metabolites formed from the condensation of two carbon units—isopentenyl diphosphate (IPP) and dimethylallyl diphosphate (DMAPP)—which are classified as plant secondary metabolites [30]. In plants, these two compounds synthesized through two separate biological pathways—the methylerythritol phosphate (MEP) and mevalonate (MVA) pathways—mainly producing precursors for monoterpenes, diterpenes, or sesquiterpenes [31,32]. Terpene synthases (TPSs) are essential enzymes for the biosynthesis of terpenoid compounds, and the diversity of terpenoids is primarily determined by the diversity of TPSs [33]. After the conversion of precursor substances, plants produce different terpenoids through the catalytic action of TPSs [34]. Most volatile terpenoids are directly formed by the catalysis of TPSs, while a portion is produced through hydroxylation, acylation, or methylation of TPS catalytic products (Figure 3A). 

DNA methylation can prevent transcription factors from binding and so block gene transcription, affecting terpenoid synthesis in plants. The gene *TPS2* is key in the linalool synthesis pathway of *Osmanthus fragrans*. Due to lower methylation levels in the *OfTPS2* promoter region of cv. ‘Chenghong Dangui’ compared to ‘Zaohuang’, the levels of linalool and its oxides are significantly higher in the petals of ‘Chenghong Dangui’ [35]. In *Rosa hybrida*, both *RhNUDX1* and *RhGDS* participate in terpenoid synthesis as well as in the response to cold stress. Under low-temperature stress, *RhNUDX1* and *RhGDS* undergo methylation, which reduces their transcript levels to decrease the release of terpenoid compounds [36].

### 3.2. Phenylpropanoids/Benzenoids

Benzenoids constitute the second largest class of plant volatile organic compounds, with the aromatic amino acid phenylalanine (Phe) as the substrate. The Phe is synthesized via two alternative pathways, linking central carbon metabolism with Phe synthesis, namely the shikimate pathway and the aromatic amino acids pathways [37]. In the presence of phenylalanine ammonia-lyase (PAL), benzenoids (C_6_–C_1_) and phenylpropanoids (C_6_–C_3_) compounds produce *trans*-cinnamic acid (CA) and vie with phenylacetaldehyde synthase for Phe utilization [38]. Subsequently, propyl side-chain of CA undergoes cleavage of two carbon atoms through the *β*-oxidation pathway, the non-*β*-oxidative pathway, or a combination of both pathways to generate benzoic acid [39]. Recently, the *β*-oxidation pathway was demonstrated in *Petunia hybrida*, which initiates with the conversion of CA into its CoA thioester form, catalyzed by cinnamoyl-CoA ligase (CNL). This is followed by a sequence of reactions including hydration, oxidation, and cleavage of the *β*-keto thioester, ultimately leading to the formation of benzoyl-CoA [40]. The non-*β*-oxidation pathway utilizes benzaldehyde dehydrogenase (BALD), which has been characterized in *Antirrhinum majus*, to oxidize benzaldehyde into benzoic acid, with benzaldehyde regarded as a crucial metabolic intermediate in this pathway, though the specific enzymes responsible for its formation have yet to be identified [41]. Finally, benzoic acid MT (BAMT) catalyzes the methylation of benzoic acid to form methyl benzoate [42]. The biosynthesis of floral phenylpropanoids (C_6_–C_3_) begins with the same initial steps as the lignin biosynthesis pathway, initiating with the formation of alcohol compounds. These compounds then undergo two enzyme-catalyzed reactions to form esters, which are further converted to the phenylpropanoids eugenol and isoeugenol by the action of eugenol and isoeugenol synthases [38]. Phenylpropanes (C_6_–C_2_), however, compete directly with PAL for Phe and undergo a series of enzymatic reactions to produce them (Figure 3B).

In plant petals, phenylpropanoid/benzenoid compounds can be utilized to enhance the synthesis of volatile compounds by direct methylation of odor precursors [29]. The gene for (iso)eugenol *O*-MT (IEMT) was the first identified Type I MT gene, and IEMT catalyzes the formation of eugenol and isoeugenol in *Clarkia breweri* [43]. The later discovered *RcOMT1* is the gene for a novel IEMT that positively regulates the biosynthesis of eugenol and isoeugenol in the petals of *Rosa chinensis* [44]. Additionally, orcinol *O*-MTs (*RcOOMT1* and *RcOOMT2*) catalyze the synthesis of 3,5-dimethoxytoluene (DMT) and 1,3,5-trimethoxybenzene (TMB) in the last three methylation steps [45]; TMB is also synthesized by phloroglucinol *O*-MT (POMT) and catalyzes the production of intermediates in the previous step, thereby releasing the rose aroma [46]. In *R. hybrida*, *RhOOMT1* and *RhOOMT2* catalyze a similar process for DMT formation [47]. Gene *EjOMT1* is also a novel member of the *O*-MTs participating in the synthesis of phenolic compounds in *Eriobotrya japonica* flowers [48]. The SABATH family represents Type III *O*-MTs, catalyzing the biosynthesis of volatile esters, including benzoic acid and salicylic acid, in plants, such as *A. majus*, *Petunia hybrida*, *Stephanotis floribunda*, and *Nicotiana tabacum* [49,50]. Benzoic acid/salicylic acid carboxyl MT (BSMT) can catalyze the formation of methyl esters of benzoic acid and salicylic acids, respectively. For example, *LiBSMT* plays a prominent role in the production and release of benzoic acid methyl ester in *Lilium brownii* cv. ‘Yelloween’ [51]. The abundant release of benzoic acid methyl ester in *Hedychium coronarium* is attributed to the coordinated and high-level expression of *HcBSMT2* and *HcCNL* in the biosynthetic pathway [52]. The p-methoxybenzoic acid carboxyl MT (MBMT), as a novel plant MT capable of catalyzing benzoic acid and JA, is mainly responsible for the unique fragrance in *Eriobotrya japonica* [53].

The phenylpropane biosynthesis pathway encompasses numerous differentially expressed genes and differentially methylated genes, playing a significant role in the synthesis of floral aroma in plants. The genes *PmCFAT1a/1c*, *PmBEAT36/37*, *PmPAL2*, *PmPAAS3*, *PmBAR8/9/10*, and *PmCNL1/3/5/6/14/17/20* were differentially methylated during flowering in *Prunus mume* and have been identified as key enzyme genes encoding its floral aroma biosynthesis, which are involved in the majority of processes within the phenylpropane biosynthetic pathway [54]. This revealed, for the first time, the critical role of DNA methylation in regulating floral scent biosynthesis.

### 3.3. Fatty Acid Derivatives

Fatty acid derivatives constitute the third class of volatile organic compounds in plants and are infrequent components of plant floral aroma. They derive from unsaturated C_18_ fatty acids, namely linoleic and linolenic. These compounds are catalyzed by lipoxygenase (LOX), which converts the octadecanoid precursors into 9- and 13-hydroperoxide intermediates that can enter two different branches of the LOX pathway for further metabolism [55]. One branch consists of allene oxide synthase (AOS) that converts the 13-hydroperoxide intermediate through a series of cyclization and reduction reactions to produce JA, which is then converted into methyl jasmonate (MeJA) by jasmonic acid carboxyl MT. The other branch involves the participation of three enzymes: hydroperoxide lyase (HPL), alcohol dehydrogenase (ADH), and alcohol acyl transferase (AAT) [56]. Under the catalysis of HPL, these two types of hydroperoxide fatty acid derivatives are converted into C_6_ and C_9_ aldehydes. Subsequently, ADH reduces them to alcohol compounds, which are then further converted into esters by AAT. These saturated and unsaturated C_6_/C_9_ aldehydes and alcohols are typically known as green leaf volatiles, imparting a unique floral scent to the petals [57] (Figure 3C).

Also a member of the SABATH family, JMT activates MeJA production in *Arabidopsis thaliana*. It is an essential enzyme in modulating the response of plants to JA [58]. In *Cymbidium faberi*, *CfbHLH* can directly interact with the promoters of *CfAOC* and *CfJMT*, promoting MeJA synthesis [59]. Additionally, it has been shown that fatty acid methyl esters may be formed by multiple unidentified MTs, which are also crucial in the biosynthesis of plant floral aroma. For example, *VcSABATH1/3* is primarily responsible for the synthesis of methyl hexanoate in *Victoria cruziana*, which emit a strong fragrance at night [60].

## 4. Impact of Methylation Modification on the Formation of Floral Color in Ornamental Plants

In plant petals, flower color is mainly determined by three key pigments: carotenoids, flavonoids, and alkaloids. Variations in the accumulation and combination of these pigments in plants can lead to a wide range of colors [61]. Carotenoids confer yellow, orange, and red hues to flowers, as observed in certain plants, like *Tagetes erecta* and *Calendula officinalis* [62,63]. Flavonoids are major players in plant flower coloration, leading to red, purple, blue, and yellow hues, as seen in *Pericallis hybrida* and *Rhododendron simsii* [64,65]. Methylation modification is one of the main modification reactions in plant pigment formation [66]. It affects the color of flower structures, such as petals and stamens, by regulating the expression of key genes, increasing the diversity and stability of colors, and playing an important regulatory role in plant pigment synthesis.

### 4.1. Carotenoids

**Figure 4:**
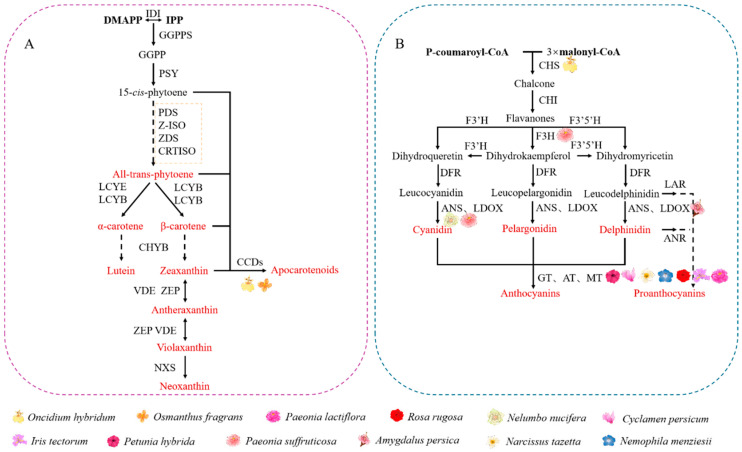
Key enzyme genes in the pathway of phytochrome metabolism in plant petals. (**A**) Diagram of structural genes involved in the regulation of carotenoid metabolism in flowers; (**B**) diagram of structural genes involved in the regulation of flavonoid metabolism in flowers.

Carotenoids belong to a group of naturally occurring, lipid-soluble compounds, typically consisting of C_40_ hydrocarbons and their oxidative derivatives [67]. In plants, carotenoids are synthesized within the plastids via the MEP pathway, generating geranyl pyrophosphate (GPP), farnesyl pyrophosphate (FPP), and geranylgeranyl pyrophosphate (GGPP). Phytoene is the first molecule with carotenoid structural characteristics, formed by the condensation of two GGPP units. Although phytoene is colorless, it is subsequently converted into red lycopene through the catalytic actions of phytoene desaturase (PDS), ζ-carotene desaturase (ZDS), ζ-carotene isomerase (ZISO), and carotene isomerase (CRTISO) [68]. Subsequently, α-carotene is transformed into lutein through cytochrome P450 carotene hydroxylase (CYP), while β-carotene produces various luteins under the catalysis of β-carotene hydrolase (BCH), zeaxanthin epoxidase (ZEP), and other hydroxylases and epoxidases in the β-branch, exhibiting bright orange and red colors [69]. Carotenoid degradation is primarily catalyzed by carotenoid cleavage dioxygenases (CCDs), generating various types of apo-carotenoids [70] (Figure 4A).

**Figure 5:**
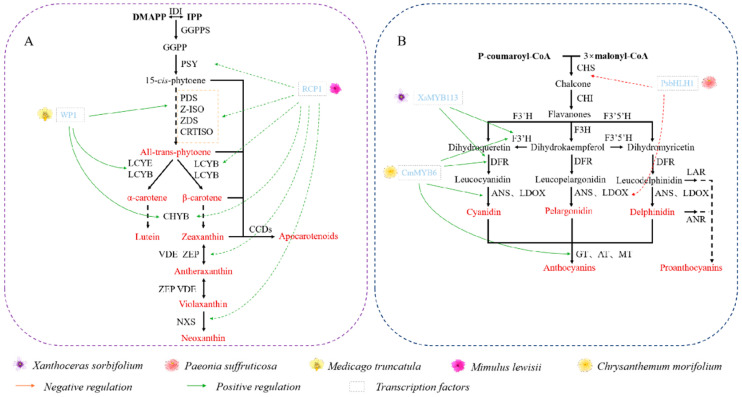
Transcription factors in the pathway of phytochrome metabolism in plant petals. (**A**) Diagram of specific transcription factors involved in the regulation of carotenoid metabolism in flowers; (**B**) diagram of specific transcription factors involved in the regulation of flavonoid metabolism in flowers.

The degree of methylation of CCD promoters often leads to differences in petal color. In *O. fragrans* ‘Chenghong Dangui’, the overall methylation rate of the upstream promoter region of *OfCCD4* is significantly higher than that of ‘Zi Yingui’, affecting expression of *OfCCD4*. This allows ‘Chenghong Dangui’ to accumulate large amounts of β-carotene, resulting in a deeper orange–red color [71]. Similarly, in *Oncidium* orchid, compared to ‘White Jade’ (white), ‘Gower Ramsey’ (yellow) has a higher level of DNA methylation in the *OgCCD1* promoter, which suppresses the transcription level of *OgCCD1* and leads to yellow coloration [72]. Methylation is also associated with the regulation of R2R3-MYB transcription factors that control carotenoid levels in petals (Figure 5A). The R2R3-MYB protein WHITE PETAL1 (WP1) regulates pigment deposition in flowers of *Medicago truncatula* by upregulating the carotenoid biosynthesis genes *MtLYCE* and *MtLYCB* [73]. The inactivation of the R2R3-MYB transcription factor REDUCED CAROTENOID PIGMENTATION1 (RCP1) can suppress expression of *PSY1*, *ZISO*, and *ZDS2*, thereby reducing carotenoid accumulation in *Mimulus lewisii* [74].

### 4.2. Flavonoids

Flavonoids are a class of polyphenolic compounds based on the benzopyrone ring, with a basic skeleton of C_6_–C_3_–C_6_ [75]. Over 5000 types of flavonoids have been identified, among which anthocyanins are the most common, and are the main water-soluble pigments responsible for plant coloration [76]. In plants, anthocyanins are typically present as glycosides, known as anthocyanidins. They include petunidin, cyanidin, delphinidin, malvidin, peonidin, and pelargonidin [77]. Petunidin and malvidin are formed by methylation of the 3′ and 5′ hydroxyl groups of cyanidin, while peonidin is formed by methylation of the 3′ hydroxyl group of delphinidin [78]. The production of anthocyanins is primarily governed by two types of genes for key enzymes in the anthocyanin synthesis pathway (Figure 4B), and transcription factors, including MYB, bHLH, and WD40 [79] (Figure 5B). During petal formation, methylation modification mainly affects flavonoid metabolism, including pigment deposition and spot formation.

#### 4.2.1. Effect on Pigment Deposition in Plant Petals

The methylation of the downstream synthesis gene ANS promoter region is a key factor causing color differences in *Nelumbo nucifera*. The ANS promoter region in the white cultivar ‘Baige’ has a higher methylation level compared to the red cultivar ‘Yehonglian,’ which inhibits its binding with transcription factors, affecting the low expression of ANS and the accumulation of anthocyanins, thereby resulting in variation in color between red and white *N. nucifera* cultivars [78]. Subsequent to anthocyanin biosynthesis is *O*-methylation and other modifications. Two pairs of anthocyanin *O*-MTs (*Mt1*/*Mt2* and *Mf1*/*Mf2*) were successfully isolated from *Petunia hybrida* [80]. The differential expression of *CkmOMT2* accounts for the distinct characteristics between the red–purple- and the purple-flowered fragrant cyclamen ‘Kaori-no-mai’. In vitro enzyme assays have shown that *CkmOMT2* catalyzes the *O*-methylation of the B-ring of anthocyanin substrates at the 3′ or 3′,5′ positions [81].

Anthocyanin *O*-MT (AOMT) is mostly associated with differences in purple or red flower traits. In *Iris tectorum*, AOMT mutations change flower color from purple to red–purple [82]. Two homologous AOMT genes from purple-flowered (*PsAOMT*) and red-flowered (*PtAOMT*) *Paeonia* plants have been identified, and *PsAOMT* exhibits stronger methylation activity in vitro compared to *PtAOMT*, resulting in the color difference [83]. In *Rosa rugosa* ‘Zi zhi,’ *RrAOMT* is methylated to produce peonidin, which in turn produces the purple flower phenotype, utilizing cyanidin as a substrate [84]. The flavonoid *O*-MT (*NtFOMT2*) exhibits a similar expression pattern to AOMT, suggesting its potential involvement in flavonoid methylation reactions in *Narcissus tazetta* [85]. Furthermore, *Nemophila menziesii* utilizes anthocyanin *O*-MT (*NmAMT6*) to catalyze the biosynthesis of anthocyanin glucosides, resulting in a blue phenotype [86]. Anthocyanin synthesis is mainly regulated by four transcription factors: MYB, bHLH, WD40, and bZIP. In *Xanthoceras sorbifolium*, *XsMYB113* is regulated by de novo methylation, which suppresses the expression of *XsF3’H* and *XsDFR*, the key genes for anthocyanin synthesis, and results in a gradient color formation at the base of the inner whorl of white petals [87]. In *Chrysanthemum morifolium*, *CmMYB6* transcription factor can positively regulate anthocyanin accumulation by binding to the downstream structural genes *CmF3’H*, *CmDFR*, *CmANS*, and *Cm3GT*. The promoter region of *CmMYB6* undergoes CHH methylation in the petals, leading to color differences between pink- and yellow-flowered plants [88]. 

#### 4.2.2. Effect on Spot Formation in Plant Petals

Currently, research on plant spots is mainly focused on transcriptional regulation mechanisms, but some studies focus on post-transcriptional regulation, such as methylation. DNA methylation typically results in the loss of the gene’s original function, which is involved in the formation of irregular-type spots. Gene *OgCHS* is crucial in the variation of spot formation in *Oncidium* spp. The hypermethylation of the 5′-upstream promoter region of *OgCHS* leads to gene silencing, resulting in the failure of anthocyanin accumulation in ‘Honey Dollp’ floral tissues, thus preventing the formation of red spots, like those in the variegated cultivar ‘Gower Ramsey’ [89]. Gene *PsbHLH1* directly suppresses the promoter activity of *PsCHS* in *Paeonia suffruticosa* ‘Shima Nishiki,’ regulating the synthesis and transport of anthocyanins, thereby exhibiting red–white bicolored traits [90]. The methylation rate of variegated flower buds (VF) in *Amygdalus persica* is similar to that of red flowers (RF), but the methylation state of the leucoanthocyanidin dioxygenase (LDOX) promoter is also significantly higher in VF compared to RF during flower stages 2 based on bisulfite sequencing PCR results. This results in lower enzyme activity and gene expression of LDOX in VF, higher anthocyanin accumulation, and the development of variegated flower buds [91]. The promoter regions of key structural genes *PrANS* and *PrF3H* in the petals of *Paeonia rockii* ‘Shusheng Pengmo’ show a highly methylated state. During petal development, methylation levels differ between the spotted and non-spotted areas, leading to the formation of spots [92].

## 5. Conclusions and Future Outlook

Methylation is an important epigenetic modification affecting ornamental traits in plants, influencing the release of plant petal volatiles and pigment deposition through the action of MTs and DMs. Methylation modifications can not only catalyze the methylation of cytosine in genetic material but can also be induced by mutagens, leading to additional methylation of genetic material, which is equally crucial for the development of both aroma and color in plant petals [23]. Moreover, as genome sequencing technologies, proteomics, and metabolomics have advanced, various types of MTs and DMs have been identified and characterized in plants [93,94]. It is of great significance to study the methylation modification reactions regulating the production of aroma substances and pigments in plant petals, for example, by exploring the regulatory mechanisms of different methylation modifications, including methylation of genetic and methylation of non-genetic material, which can better reveal the role of methylation reactions in modifying aroma and color in plant petals. Identifying key enzyme genes involved in methylation pathways can further elucidate the metabolic mechanisms of color and aroma synthesis in ornamental plants from an epigenetic perspective. This provides genetic resources for using gene editing technologies to improve plant color and aroma. Inducing the loss of function in targeted MTs and DMs related to floral scent synthesis and pigment metabolism can offer new insights into the regulation mechanisms of volatile compounds and pigment ratios, as well as the processes of changes in floral aroma and color components in ornamental plants.

The regulatory mechanisms of methylation modifications in plants have consistently been a prominent area of research, yet studies on modifying odor and color molecules through MTs and DMs are limited. Additionally, research on methylation modifications affecting flower color and aroma primarily focuses on the methylation of non-genetic substances, with in-depth studies on epigenetic modifications still lacking. There has been substantial research on the formation mechanisms of epigenetic modifications, such as DNA methylation and histone methylation, which are also relatively well-studied in aspects affecting plant growth and development (fruit development and ripening) through the dynamic regulation of MTs and DMs [95]. However, the potential mechanisms of demethylation-induced gene silencing are still not well understood. Thus, the mechanisms by which heritable modifications, like DNA methylation and histone methylation, affect floral scent and color warrant further exploration. Furthermore, whether MTs or methylation pathways discovered in specific species function in identical or comparable regulatory roles in other plant species remains an important direction for research. Increasing numbers of studies demonstrate a potential correlation between plant aroma and color, indicating that they may share biochemical pathways—terpenoids and carotenoids are both produced by the MEP biosynthetic pathway [96], and phenyl/phenylpropanoid production and anthocyanin synthesis are both branches of the shikimate pathway that share a common precursor, Phe [97]. However, how methylation modifications trigger color-fragrance combination patterns are not yet clear. Although some transcription factors and enzyme genes have been confirmed as crucial regulators of the color and aroma pathways, whether they undergo methylation and how methylation modifications occur still merit investigation, especially how promoter methylation activates gene transcription and triggers changes in color and aroma.

## Data Availability

All data in this study can be found in the manuscript.
